# A Retrospective Paired Comparison Between Untargeted Next Generation Sequencing and Conventional Microbiology Tests With Wisely Chosen Metagenomic Sequencing Positive Criteria

**DOI:** 10.3389/fmed.2021.686247

**Published:** 2021-10-06

**Authors:** Hanyu Qin, Jinmin Peng, Ling Liu, Jing Wu, Lingai Pan, Xiaobo Huang, Man Huang, Haibo Qiu, Bin Du, The China Critical Care Clinical Trials Group (CCCCTG)

**Affiliations:** ^1^State Key Laboratory of Complex, Severe and Rare Disease, Medical ICU, Peking Union Medical College Hospital, Peking Union Medical College and Chinese Academy of Medical Sciences, Beijing, China; ^2^Department of Critical Care Medicine, School of Medicine, Zhongda Hospital, Southeast University, Nanjing, China; ^3^Department of Intensive Care Unit, The Second Affiliated Hospital, Zhejiang University School of Medicine, Hangzhou, China; ^4^Department of Critical Care Medicine, Sichuan Provincial Hospital, University of Electronic Science and Technology of China, Chengdu, China; ^5^Sichuan Translational Medicine Research Hospital, Chinese Academy of Sciences, Chengdu, China

**Keywords:** metagenomic sequencing, pathogen detection, lower respiratory tract infection, conventional microbiology tests, positive criteria

## Abstract

**Objectives:** To evaluate the performance of metagenomic next generation sequencing (mNGS) using adequate criteria for the detection of pathogens in lower respiratory tract (LRT) samples with a paired comparison to conventional microbiology tests (CMT).

**Methods:** One hundred sixty-seven patients were reviewed from four different intensive care units (ICUs) in mainland China during 2018 with both mNGS and CMT results of LRT samples available. The reads per million ratio (RPM_sample_/RPM_non−template−control_ ratio) and standardized strictly mapped reads number (SDSMRN) were the two criteria chosen for identifying positive pathogens reported from mNGS. A McNemar test was used for a paired comparison analysis between mNGS and CMT.

**Results:** One hundred forty-nine cases were counted into the final analysis. The RPMsample/RPM_NTC_ ratio criterion performed better with a higher accuracy for bacteria, fungi, and virus than SDSMRN criterion [bacteria (RPMsample/RPM_NTC_ ratio vs. SDSMRN), 65.1 vs. 55.7%; fungi, 75.8 vs. 71.1%; DNA virus, 86.3 vs. 74.5%; RNA virus, 90.9 vs. 81.8%]. The mNGS was also superior in bacteria detection only if an SDSMRN ≥3 was used as a positive criterion with a paired comparison to culture [SDSMRN positive, 92/149 (61.7%); culture positive, 54/149 (36.2%); *p* < 0.001]; however, it was outperformed with significantly more fungi and DNA virus identification when choosing both criteria for positive outliers [fungi (RPMsample/RPM_NTC_ ratio vs. SDSMRN vs. culture), 23.5 vs. 29.5 vs. 8.7%, *p* < 0.001; DNA virus (RPMsample/RPM_NTC_ ratio vs. SDSMRN vs. PCR), 14.1 vs. 20.8 vs. 11.8%, *p* < 0.05].

**Conclusions:** Metagenomic next generation sequencing may contribute to revealing the LRT infection etiology in hospitalized groups of potential fungal infections and in situations with less access to the multiplex PCR of LRT samples from the laboratory by choosing a wise criterion like the RPMsample/RPM_NTC_ ratio.

## Introduction

Lower respiratory tract (LRT) infection is a common cause of intensive care unit (ICU) admission. The Management of Severe sepsis in Asia's Intensive Care unitS (MOSAICS) study revealed that among 1,285 severe sepsis patients admitted to Asian ICUs, 37.4% of the sources of infection were attributed to the lungs (1). Meanwhile, acute respiratory infection often leads to an unfavorable outcome, with in-hospital mortality increasing by 21.8% over a decade in one French region (2). Indeed, a point prevalence study of ICU infection, which recruited 1,150 centers, reported that less than two-thirds (5,259/8,135, 65%) of the ICU patients with probable or definite infections received at least one positive culture (3). Even combining cultures, targeted molecular methods and serology all together to routinely investigate lower respiratory tract (LRT) infection microbial etiology, Leven et al. found out that the potential pathogen detection rate was only 59% (1,844/3,104) in this study (4). Approximately 40% of the cases were causative agents undetermined by conventional microbiological tests (CMT).

Next generation sequencing, as a high-throughput technique, with a cost that was largely reduced since 2004 (5), is now widely used in clinical metagenomics, specifically for unbiased pathogen detection. In particular, metagenomic next generation sequencing (mNGS) allows for the novel findings and taxonomical classification of microorganisms, which could tremendously impact infectious disease diagnosing (6).

Large sample size studies evaluating mNGS diagnostic performance compared with CMTs either as a first-line test or the last resort in clinical scenarios were conducted. Parize et al. designed a prospective study with a cohort of 101 patients and a 30-day follow-up. They reported that untargeted next generation sequencing had a high negative predictive value of 98.4% (95% CI 95.3–100%) in bacteria and viruses, but with a restriction on the identification of fungi or parasites (7). Xing et al. claimed in his 213 case series that the mNGS detection rate was 57%, while in the laboratory of Chiu, mNGS succeeded in 32 central nervous system (CNS) infection diagnoses from 204 patients. The performance of mNGS remains controversial, and studies in LRT specimens were limited (8, 9).

A multicentered research was conducted with patients with suspected LRT infections, and both mNGS and CMT results reported from LRT samples, which aimed to interpret mNGS results in a fair way for common infectious agents.

## Methods

### Study Design

One hundred sixty-seven patients admitted to four different ICUs in mainland China during 2018 were retrospectively studied. These cases were reviewed considering the following inclusion criteria: (i) patients with respiratory symptoms requiring oxygen therapy or any other organ support in the ICU, (ii) patients with radiology images (chest x-ray or CT scanning) showing lung infiltration, (iii) patients with a suspected infection causing the conditions described meeting criteria (i) and (ii), and (iv) patients with lower respiratory tract samples collected for both mNGS and CMT.

### Data Collected From Medical Records

Demographic information, comorbidities, immune state, disease severity upon ICU admission, duration between ICU admission and attempt to sequence LRT specimen, ICU intervention, clinical outcome, and conventional microbiological test results were all collected from the medical record of each patient through the hospital information system. Disease severity was assessed using an Acute Physiology and Chronic Health Evaluation (APACHE) II score (10) and a Sequential Organ Failure Assessment (SOFA) score (11). A higher score indicates more severe clinical presentation. Furthermore, ICU intervention in this study was listed but not limited to vasopressor utilization, invasive or non-invasive mechanical ventilation, and continuous renal replacement therapy. Clinical outcome was illustrated as the length of stay in the ICU and ICU mortality.

### Retrieving the LRT Sample and CMT Positive Agreement on Clinically Significant Microbes

Lower respiratory tract samples such as sputum, trachea aspirates, or bronchoalveolar lavage fluids were investigated in this study. The available CMTs of LRT samples (smear, culture, and multiplex PCR) in participating centers and significant pathogen consideration were consistent with previous publication (Supplementary Tables 1, 2) (12). Microbes detected *via* CMT were identified as true pathogens rather than commensals or contaminants. Serum antigen test results were also reviewed, while serum antibody test results were excluded due to a failure to distinguish acute infection. More specifically, immunoglobulin G (IgG) antibodies reported in our cohort lacked track of a 4-fold rise while IgM antibody test results were not recommended for the infection diagnoses of adenovirus, *Mycoplasma pneumoniae*, and *Legionella/Chlamydia* spp. (13, 14).

### Library Construction, Sequencing, Bioinformatic Analysis, and Criteria for a Positive Result

After extraction, nucleic acid fragments underwent end repairing, adapter ligation, and amplification to construct the library, quality control of which was assessed by Agilent 2100 Bioanalyzer (Agilent Technologies, Santa Clara, CA, United States). The qualified library was sequenced on the BGISEQ-100/50 platform. A non-template specimen was parallelly run for the purpose of contamination control (non-template control, NTC).

Raw sequencing data were filtered by removing low-quality reads, and clean reads were mapped to the human reference genome (hg19) to subtract the host portion by Burrows-Wheeler Alignment. The remaining reads were classified by simultaneously mapping to four microbial genome databases, composed of 4,152 whole genome sequences of viral taxa, 3,446 bacteria, 206 fungi, and 140 parasites associated with human disease (15).

The mapped reads number of each microbe in each respiratory sample was normalized in three ways (15, 16).

(i) Mapped reads abundance relative to other microbes in the same sample:
  $$\begin{array}{l} \text{Reads per million }\left(\text{RPM}\right)=\frac{\text{Mapped reads number}\left(\text{MRN}\right)\;*\;10^{6}}{\text{Total sequencing reads}} \end{array}$$
(ii) Mapped reads abundance relative to the same microbe at the species level in other samples of this study cohort using Z-score.
  $$\begin{array}{l} \text{Z}-\text{score}=\frac{\chi -\mu \;}{\sigma }\; \end{array}$$
  χ is the log10 transformation of RPM of one microbe. μ is the mean of log_10_RPM of the same microbe in this study cohort. σ is the SD of log_10_RPM of the same microbe in this study cohort.
(iii) Uniquely mapped reads in this study were described as stringently mapped reads number (SMRN) and normalized as SDSMRN.
  $$\begin{array}{l} \text{SDSMRN}=\frac{\text{SMR}{\text{N}}^{*}20^{*}10^{6}}{\text{Total sequencing reads}} \end{array}$$

The positive criteria for the mNGS result were set as follows, which were consistent with literature reviews at the species level and those that found that microbes were evidenced of pathogenicity in the lungs (15–17). A value of “1” was adjusted to the RPM of the non-template control with no pathogenic bacteria, fungi, or viruses (18).

(i) RPMsample/RPM_NTC_ ratio ≥10, or SDSMRN ≥3 (mycobacteria excluded) for bacteria
(ii) RPMsample/RPM_NTC_ ratio ≥1, or SDSMRN ≥3 for fungi/DNA virus
(iii) RPMsample/RPM_NTC_ ratio ≥1, or SDSMRN ≥1 for RNA virus
(iv) SDSMRN ≥100 for parasites
(v) SDSMRN ≥3 for *Mycoplasma*/*Chlamydia* spp.
(vi) SDSMRN ≥1 (or SDSMRNG ≥1 at genus level) for *Mycobacterium tuberculosis* (MTB) complex
(vii) Reported *Nocardia* spp. by mNGS all considered positive

### Statistical Analysis

A Mann–Whitney *U*-test was used for non-parametric data analysis. A 2 × 2 contingency table was drawn to calculate sensitivity, specificity, positive predictive value (PPV), and negative predictive value (NPV). Data were reported as absolute values with 95% CI (7). The marginal frequencies of the 2 × 2 table were tested by a McNemar test. Significance was considered when *p* < 0.05. The SPSS 22.0 software was used.

## Results

### Patient Characteristics and General Sequencing Information

One hundred forty-nine cases were enrolled into the final analysis since the total sequencing reads in the mNGS reports of 18 patients were missing, making both RPM and SDSMRN infeasible to calculate. Among these 149 patients [median age 59, interquartile range (IQR) from 47.5 to 70; 104 (69.8%) male], 75 (50.3%) were at immunocompromised states. Fifty (33.6%) were prescribed prolonged corticosteroids (defined as a minimum dose of 0.3 mg/kg/d of prednisone or an equivalent for 3 weeks at least) (12), constructing the therapy of autoimmune disease [37 (24.8%)] and post-transplantation [8 (5.4%)] or other. Twenty-one (14.9%) were receiving chemotherapy for cancer, leukemia, or lymphoma. The median APACHE II and sofa scores were 20 (IQR from 15 to 24) and 9 (IQR from 6 to 11), respectively. The median ICU stay was 12 days (IQR from 7 to 21.5 days), and ICU mortality was 51%. The LRT samples of patients were retrieved and sent for mNGS 1 day (IQR from 1 to 3 days) after ICU admission (Table 1).

**Table 1:** Patient characteristics and clinical outcome.

**Characteristic**	**Total patients (n = 149)**
Age (year), median (IQR)	59 (47.5, 70)
Male, *n* (%)	104 (69.8%)
**Disease severity**	
APACHE II score, median (IQR)	20 (15, 24)
SOFA score, median (IQR)	9 (6, 11)
**ICU intervention**	
Vasopressor, *n* (%)	83 (55.7%)
Invasive mechanical ventilation, *n* (%)	122 (81.9%)
CRRT, *n* (%)	27 (18.1%)
**Duration between mNGS test**	1 (1, 3)
**and admission (day)**	
**Clinical outcome**	
ICU death, *n* (%)	76 (51.0%)
ICU LOS (day), median (IQR)	12 (7, 21.5)
**Immunocompromised state**	**75 (50.3%)**
**Comorbidity**	
Autoimmune disease	37 (24.8%)
Hematologic malignancy	16 (10.7%)
Cancer	5 (3.4%)
Solid organ transplantation	8 (5.4%)

*IQR, interquartile range; APACHE, acute physiology and chronic health evaluation; SOFA, sequential organ failure assessment; ICU, intensive care unit; CRRT, continuous renal replacement therapy; LOS, length of stay*.

**Figure 1:**
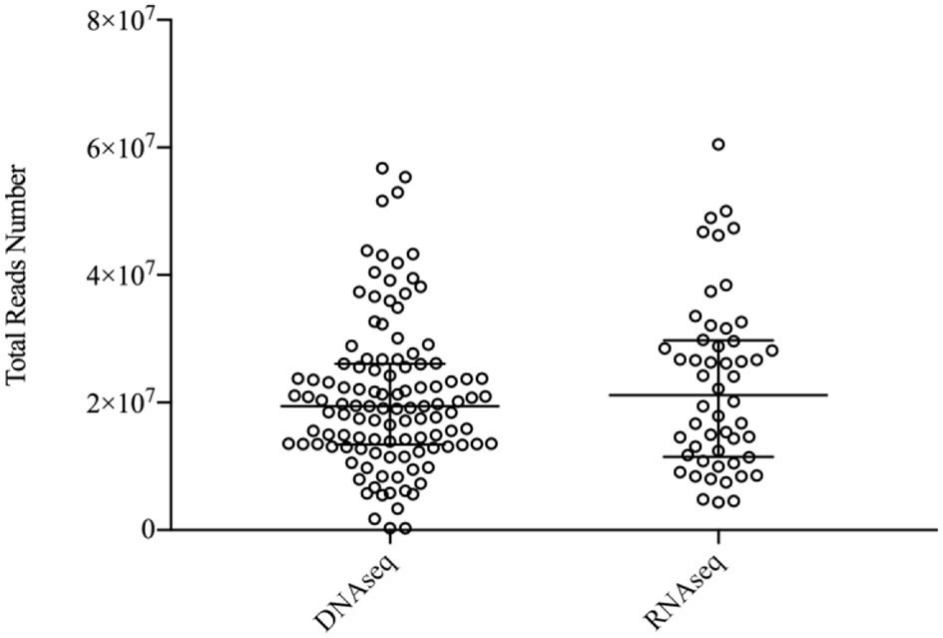
Total sequencing reads of the reports of a total of 149 patients counted to the final analysis.

**Figure 2:**
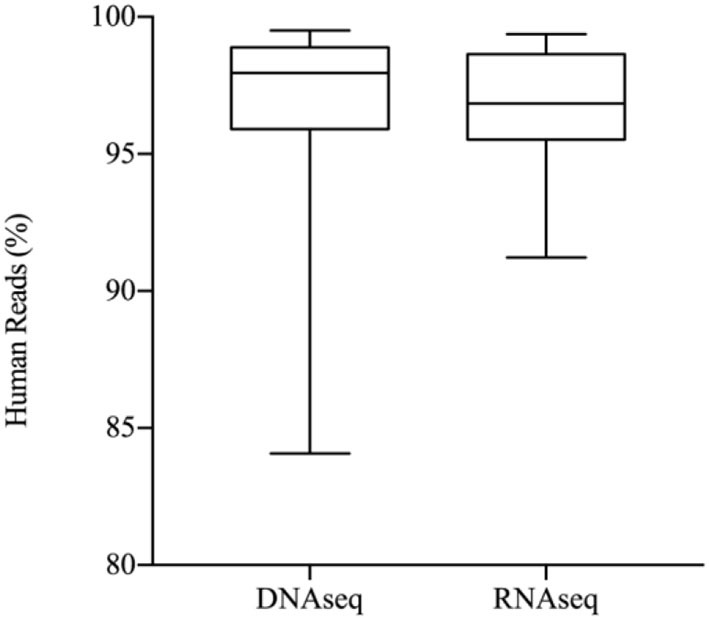
Host background of sequencing data.

In the metagenomic analysis of these 149 samples, 96 were tested by DNA sequencing (DNAseq), 35 were tested by RNA sequencing (RNAseq), and 18 were tested by DNAseq and RNAseq parallelly. No significant difference was found between the mean total yield of DNAseq and RNAseq (21,261,618 vs. 22,862,055 reads, *p* = 0.49) and for human proportion (97.1 vs. 96.8%, *p* = 0.09) (Figures 1, 2).

### Comparison of mNGS and CMT in Pathogen Detection

#### Positive mNGS Results Fulfilling RPM (Sample/NTC) Ratio Criterion

**Figure 3:**
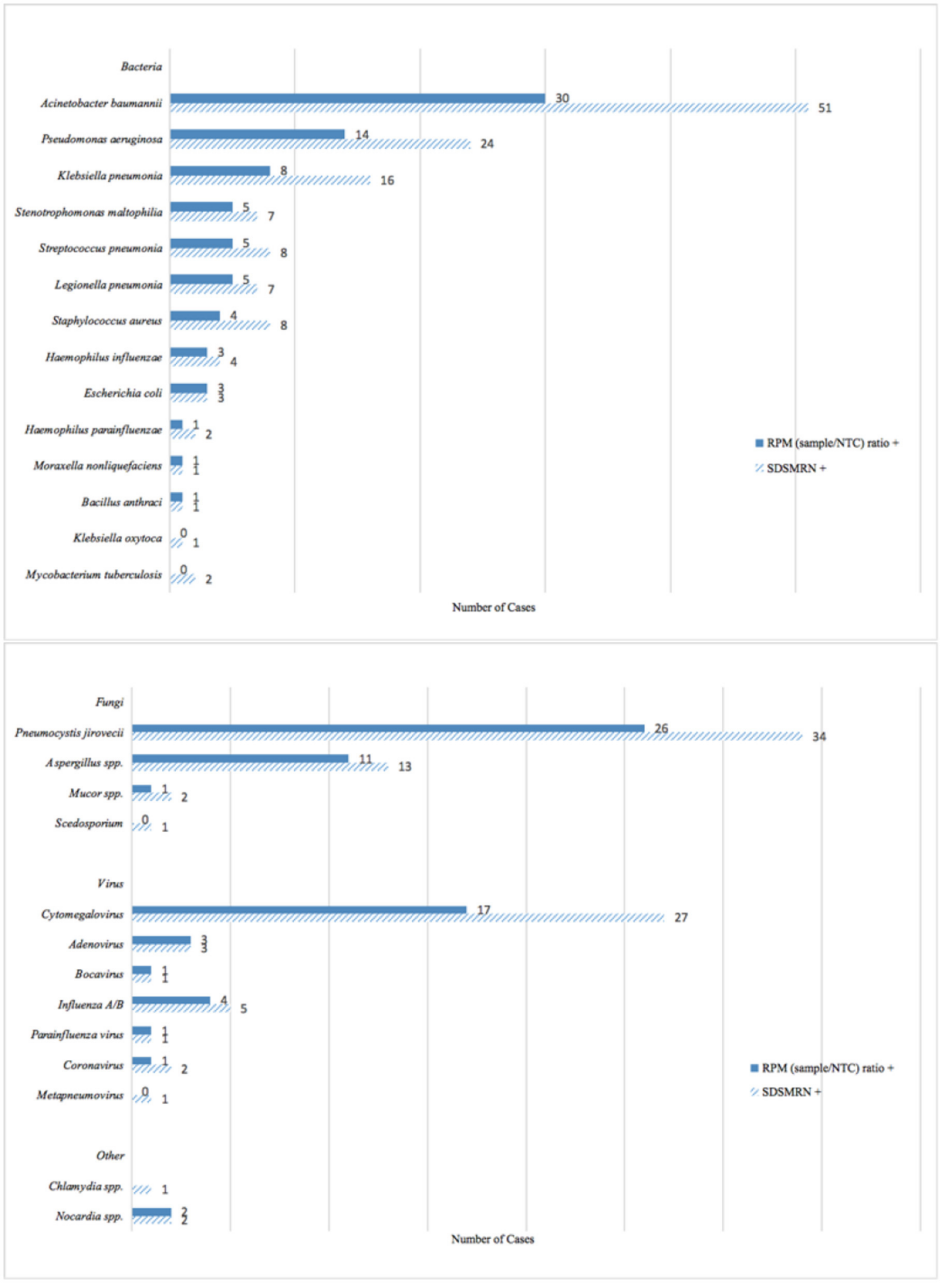
Pathogen distribution according to metagenomic sequencing positive criteria of the reports of a total of 149 patients counted to the final analysis.

**Figure 4:**
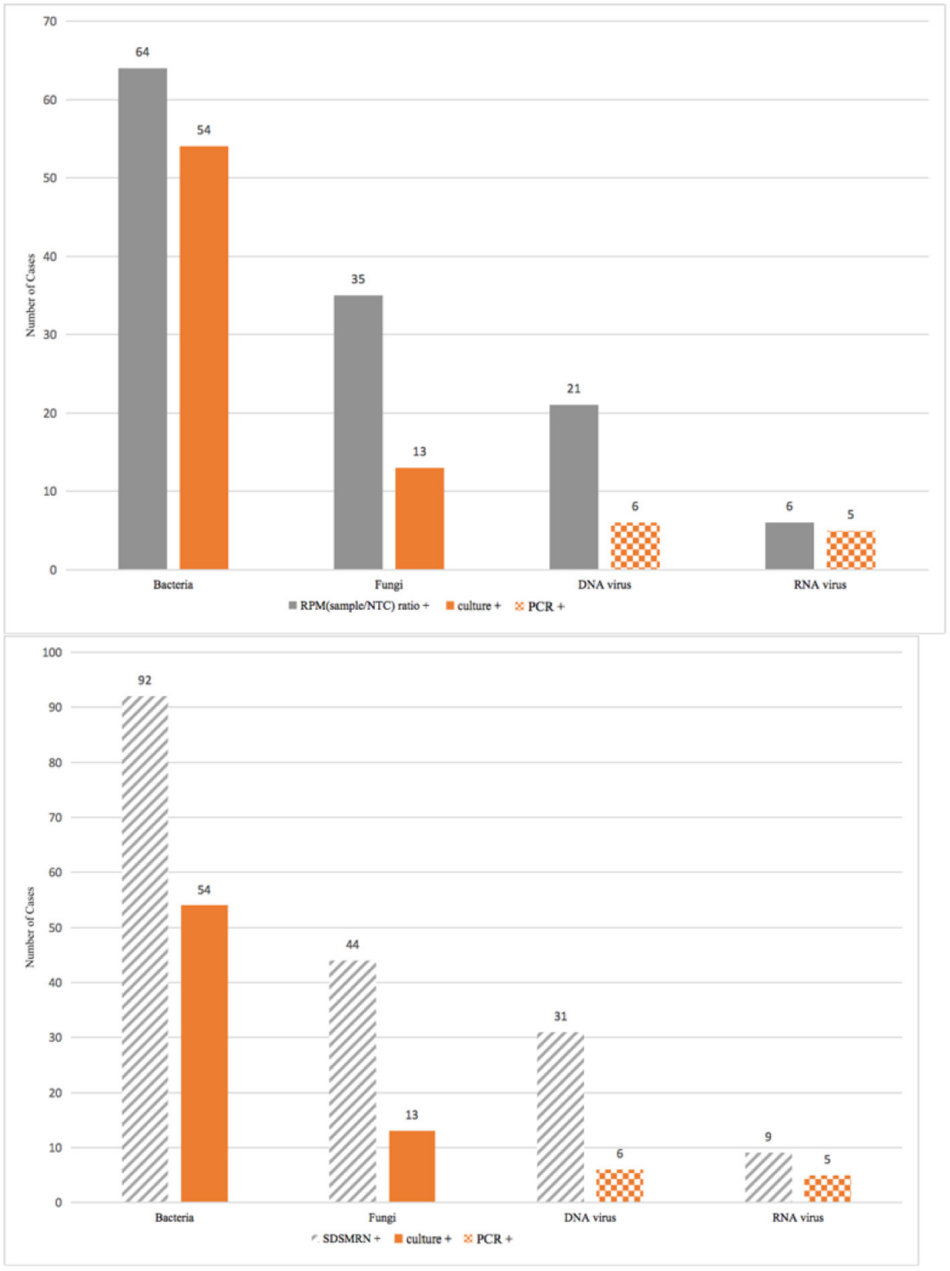
Positive cases identified by metagenomic sequencing or conventional microbiology tests (culture/PCR) from a total of 149 patients counted to the final analysis.

Bacteria were identified in 64 (64/149, 43%) cases by an RPMsample/RPM_NTC_ ratio ≥10, with the top 3 being *Acinetobacter baumannii* (30/149, 20.1%), *Pseudomonas aeruginosa* (14/149, 9.4%), and *Klebsiella pneumonia* (8/149, 5.4%). Of the 149 cases, 54 (54/149, 36.2%) were bacterial culture positive. The bacterium detection rate of mNGS seemed higher, but no significant difference was found in the paired comparison with culture by the McNemar test (*p* = 0.212) (Figures 3, 4).

Thirty-five (35/149, 23.5%) cases met the criterion of having an RPMsample/RPM_NTC_ ratio ≥1 for fungi detection, while culture suggested less (13/149, 8.7%) fungal infection (*p* < 0.001). The detection rate of the culture itself for *Aspergillus* spp. was the same as that of mNGS if choosing RPM ratio criterion (11/149, 7.4%), while the probable diagnosis of invasive pulmonary aspergillosis (IPA) would be made in 46 (46/149, 30.9%) cases using comprehensive CMTs (either culture *Aspergillosis* spp. positive or serum/bronchoalveolar lavage fluid galactomannan positive). The mNGS was not superior to comprehensive CMTs for diagnosing IPA (*p* < 0.001) (Figures 3, 4).

#### Pathogen Identified by SDSMRN Criterion and Comparison of Two Criteria

The mNGS detected 92 (92/149, 61.7%) bacterium cases with the fulfilment of SDSMRN ≥3. The SDSMRN criterion identified ~1.5 times more bacterium cases than the RPM ratio criterion, also with a better bacterium identification rate than culture (*p* < 0.001). More fungal infections were suggested by SDSMRN ≥3 (44/149, 29.5%) than RPMsample/RPM_NTC_ ratio ≥1. The detection rate of mNGS for fungi was higher than culture with either criterion chosen in the paired analysis of 149 patients (SDSMRN vs. culture, *p* < 0.001; RPM ratio vs. culture, *p* < 0.001) (Figure 4).

Meanwhile, the SDSMRN criterion had a higher sensitivity but lower accuracy than the RPM ratio criterion for both bacteria [sensitivity (SDSMRN vs. RPM ratio), 61.1 vs. 74.1%; accuracy, 65.1 vs. 55.7%] and fungi [sensitivity (SDSMRN vs. RPM ratio), 53.8 vs. 46.2%; accuracy, 71.1 vs. 75.8%] identification. The negative predictive value of mNGS in the cohort was over 75% regardless of choosing either criterion [bacteria (SDSMRN vs. RPM ratio), 75.3 vs. 75.4%; fungi, 94.3 vs. 93.9%]. The accuracy of mNGS in detecting fungi was higher than bacteria, which was also criteria independent (Table 2).

**Table 2:** Performance of metagenomic sequencing for bacterium and fungus detection.

	**Bacteria culture**					**Fungi culture**				
	**Sensitivity**	**Specificity**	**PPV**	**NPV**	**Accuracy**	**Sensitivity**	**Specificity**	**PPV**	**NPV**	**Accuracy**
RPM_sample_/RPM_NTC_ ratio ≥ 10/1	61.1% (46.9–73.8%)	67.4% (56.9–76.4%)	51.6% (38.8–64.1%)	75.3% (64.5–83.7%)	65.1% (57.4–72.8%)	46.2% (20.4–73.9%)	78.7% (70.7–85.0%)	17.1% (7.2–34.3%)	93.9% (87.3–97.3%)	75.8% (69.0–82.7%)
SDSMRN ≥ 3	74.1% (60.1–84.6%)	45.3% (35.1–55.8%)	43.5% (33.3–54.2%)	75.4% (62.0–85.5%)	55.7% (47.7–63.7%)	53.8% (26.1–79.6%)	72.8% (64.4–79.9%)	15.9% (7.2–30.7%)	94.3% (87.5–97.7%)	71.1% (63.9–78.4%)

*NPV, negative predictive value; PPV, positive predictive value*.

The SDSMRN criterion identified 13 (13/149, 8.7%) aspergillosis positive and was also not superior to comprehensive CMTs (*p* < 0.001). The accuracy of the SDSMRN criterion in identifying *Aspergillus* spp. was the same as that of the RPM ratio criterion (89.3%, 95% CI 84.3–94.2%), but the SDSMRN criterion had a higher sensitivity (36.4%, 95% CI 12.4–68.4%) if comparing mNGS with culture (Figure 3, Table 3).

**Table 3:** Diagnostic performance of metagenomic sequencing for invasive pulmonary aspergillosis.

	***Aspergillus*** **spp. culture**					**Culture or Serum/BAL fluid galactomannan**				
	**Sensitivity**	**Specificity**	**PPV**	**NPV**	**Accuracy**	**Sensitivity**	**Specificity**	**PPV**	**NPV**	**Accuracy**
RPM_sample_/RPM_NTC_ ratio ≥ 1	27.3% (7.3–60.7%)	94.2% (88.5–97.3%)	27.3% (7.3–60.7%)	94.2% (88.5–97.3%)	89.3% (84.3–94.2%)	15.2% (6.8–29.5%)	96.1% (89.8–98.7%)	63.6% (31.6–87.6%)	71.7% (63.3–78.9%)	71.1% (63.3–78.9%)
SDSMRN ≥ 3	36.4% (12.4–68.4%)	93.5% (87.6–96.8%)	30.8% (10.4–61.1%)	94.9% (89.3–97.7%)	89.3% (84.3–94.2%)	17.4% (8.3–32.0%)	95.1% (88.5–98.2%)	61.5% (32.3–84.9%)	72.1% (63.6–79.2%)	71.1% (63.9–78.4%)

*NPV, negative predictive value; PPV, positive predictive value*.

The SDSMRN criterion also suggested the possible infection of *M. tuberculosis* (*n* = 2), scedosporium (*n* = 1), metopneumovirus (*n* = 1), and *Chlamydia* spp. (*n* = 1), which would not be revealed by the RPM ratio criterion (Figure 3).

### Identification of Pathogens in Culture Negative Samples by mNGS

*Streptococcus pneumonia* (if using RPMsample/RPM_NTC_ ratio ≥10 criterion, *n* = 5; while using SDSMRN ≥3 criterion, *n* = 8), *Haemophilus influenzae, Legionella pneumonia*, and *Bacillus anthraci* were only detected by mNGS other than bacterium culture. Forty-eight (48/149, 32.2%) samples were sent for the *L. pneumonia* PCR, and only one (1/48, 2.1%) case tested positive. Of the 48 cases with a paired comparison between mNGS and Legionella PCR, both criteria also identified only one legionella positive case, which consisted of the PCR result (Figure 3).

Due to the inability of culturing *Pneumocystis jirovecii* (19), a gomori methenamine stain or PCR was used for the *P. jirovecii* pneumonia diagnosis. Fifty-eight (58/149, 38.9%) patients had both their gomori methenamine stain and PCR results reported, among which 25 (25/58, 43.1%) cases were *P. jirovecii* PCR positive, while the gomori methenamine stains only made 7 (7/58, 12.1%) *P. jirovecii* pneumonia confirmations. The RPMsample/RPM_NTC_ ratio ≥1 suggested 26 (26/149, 17.4%) *P. jirovecii* pneumonia cases with both sensitivity and accuracy [sensitivity (95% CI), 80% (58.7–92.4%); accuracy (95% CI), 91.4% (84.2–98.6%)] slightly lower than that of SDSMRN ≥3 [sensitivity (95% CI), 96% (77.7–99.8%); accuracy (95% CI), 96.6% (91.9–100%)], which identified 34 (34/149, 22.8%) *P. jirovecii* positive cases. When comparing mNGS and CMT in the paired 58 cases, mNGS performed no better than a simplex PCR but beat the gomori methenamine stain for *P. jirovecii* pneumonia diagnosis with either positive criterion chosen (RPM ratio vs. stain, *p* < 0.001; SDSMRN vs. stain, *p* < 0.001) (Figure 3, Table 4).

**Table 4:** Diagnostic performance of metagenomic sequencing for *Pneumocystis jirovecii* pneumonia.

	***Pneumocystis jirovecii*** **PCR**					**Gomori methenamine stain**				
	**Sensitivity**	**Specificity**	**PPV**	**NPV**	**Accuracy**	**Sensitivity**	**Specificity**	**PPV**	**NPV**	**Accuracy**
RPM_sample_/RPM_NTC_ ratio ≥ 1	80% (58.7–92.4%)	100% (87.0–100%)	100% (80.0–100%)	86.8% (71.1–95.1%)	91.4% (84.2–98.6%)	100% (56.1–100%)	74.5% (60.1–85.2%)	35% (16.3–59.1%)	100% (88.6–100%)	77.6% (66.9–88.3%)
SDSMRN ≥ 3	96% (77.7–99.8%)	97.0% (82.5–99.8%)	96% (77.7–99.8%)	97.0% (82.5–99.8%)	96.6% (91.9–100%)	100% (56.1–100%)	64.7% (50.0–77.2%)	28% (12.9–49.6%)	100% (87.0–100%)	69.0% (57.1–80.9%)

*NPV, negative predictive value; PPV, positive predictive value*.

Viral etiology was revealed only in one-third (54/149, 36.2%) of the patients in this study when using a multiplex PCR. Viruses of the PCR interests were limited to cytomegalovirus (CMV), influenza A/B, adenovirus, rhinovirus, respiratory syncytial virus (RSV), metapneumovirus, and parainfluenza virus. Because the samples of 96 patients only underwent a metagenomic DNAseq analysis, from which RNA viruses would be impossibly discovered, the accuracy of mNGS was evaluated for DNA virus and RNA virus cases separately. Both an RPMsample/RPM_NTC_ ratio ≥1 (21/149, 14.1%) and an SDSMRN ≥3 (31/149, 20.8%) identified more DNA virus cases than the PCR (6/51, 11.8%). The mNGS was superior to the PCR detecting DNA viruses after a paired comparison between the two criteria and the multiplex PCR of the 51 cases (RPM ratio vs. PCR, *p* = 0.007; SDSMRN vs. PCR, *p* < 0.001). Six (6/53, 11.3%) RNA viral infections were suggested by the RPM ratio criterion and nine (9/53, 17.0%) by SDSMRN ≥1. The PCR revealed less cases (5/46, 10.9%) but without significant difference compared with mNGS (RPM ratio vs. PCR, *p* = 0.5; SDSMRN vs. PCR, *p* = 0.313) (Figure 4).

Metagenomic next generation sequencing presented a sensitivity of 100% (95% CI, 51.7–100%) for DNA viruses and a negative predictive value of over 90% for RNA viruses. The mNGS detection accuracy of RNA viruses [RPM ratio (95% CI), 90.9% (78.9–100%); SDSMRN (95% CI), 81.8% (65.7–97.9%)] was higher than that of DNA virus [RPM ratio (95% CI), 86.3% (76.8–95.7%); SDSMRN (95% CI), 74.5% (62.5–86.5%)]. Still, the RPM ration criterion performed better than the SDSMRN criterion in identifying virus cases, with higher accuracy (Table 5).

**Table 5:** Performance of metagenomic sequencing for virus detection.

	**DNA virus PCR**					**RNA virus PCR**				
	**Sensitivity**	**Specificity**	**PPV**	**NPV**	**Accuracy**	**Sensitivity**	**Specificity**	**PPV**	**NPV**	**Accuracy**
RPM_sample_/RPM_NTC_ ratio ≥ 1	100% (51.7–100%)	84.4% (69.9–93.0%)	46.2% (20.4–73.9%)	100% (88.6–100%)	86.3% (76.8–95.7%)	75% (21.9–98.7%)	94.4% (70.6–99.7%)	75% (21.9–98.7%)	94.4% (70.6–99.7%)	90.9% (78.9–100%)
SDSMRN ≥ 3/1	100% (51.7–100%)	71.1% (55.6–83.2%)	31.6% (13.6–56.5%)	100% (86.7–100%)	74.5% (62.5–86.5%)	75% (21.9–98.7%)	83.3% (57.7–95.6%)	50% (13.9–86.1%)	93.8% (67.7–99.7%)	81.8% (65.7–97.9%)

*NPV, negative predictive value; PPV, positive predictive value*.

The bocavirus [*n* = 1/1 (RPM ratio positive/SDSMRN positive)] and coronavirus (*n* = 1/2) were the two novel viruses reported by mNGS outranging permits of the multiplex PCR in this cohort (Figure 3).

## Discussion

Microbiological etiology was only revealed in 12.7% of the hospitalized patients with pneumonia diagnoses in mainland China by CMTs (20). The mNGS was seen as a promising tool for its broad-spectrum and unbiased pathogen detection, with a sensitivity increase of 15% in diagnosing infection compared to culture (21). Our study first used the RPM ratio and SDSMRN as criteria for the positive identification of pathogens reported by mNGS in such a large-scale cohort of LRT infections. We also paired compared mNGS with the CMT of LRT samples in terms of their performance in detecting causative microbes. The RPM ratio criterion performed better, with a higher accuracy in identifying bacteria, fungi, and viruses, than the SDSMRN criterion in this cohort. The mNGS was only superior in bacteria detection if using SDSMRN ≥3 as the positive criterion with a paired comparison with culture but outperformed with significantly more fungi and DNA virus identification by choosing both criteria.

The mNGS test has been lacking a unified criterion for reporting clinically significant microbes due to varied sequencing instruments, the bioinformatic analysis pipeline, and the unstandardized productivity and quality control of each platform. Some studies defined mNGS positive as high-ranking microbes by sequencing abundance relative to other microbes in the same sample, probably with the purpose of balancing host background (21, 22). The human proportion of the total sequencing yield from an upper respiratory sample like an oropharyngeal swab [median (IQR), 5.1% (1.1–39.1%)] was clearly less than that of an LRT sample, which might result in more reads left for microbe mapping (23). However, a case series also proved absent microbes of the top 10 pathogens listed by mNGS in the validation run of nanopore sequencing with much longer read length, which also had a recognized 96.6% sensitivity for pathogen detection in LRT samples (24, 25). The evaluation of others for mNGS using absolute values as criteria like SMRN or unique reads number remained controversial since the difficulty of extracting nuclei acids varies from species to species and the total sequencing yield varies from sample to sample (26). It was believed that RPM could be an adequate criterion for mNGS because of its documented success in maximizing specificity for bacteria and sensitivity for fungi or viruses among immunocompromised children with pulmonary infections (18). A study by Zinter also used a combination with a Z-score ≥2 to set thresholds, which was, unfortunately, not applicable for this study. A Z-score ≥2 left eight mNGS-positive cases (8/149, 5%) in the study population (Supplementary Figure 1). The RPM to RPM (sample/NTC) ratio was also adjusted for the subtraction of contaminants to the platform. The RPM ratio criterion and the SDSMRN criterion, which was reported with a decent sensitivity (95%), were compared, detecting respiratory bacteria and fungi by Qian et al. (27).

The mNGS sensitivity for fungi detection was similar to a study by Li, which also made paired comparisons between mNGS and culture, while the mNGS of this study achieved higher specificity by wisely choosing a criterion with RPMsample/RPM_NTC_ ratio ≥1 (28). Consistent with the previous research results, mNGS also did not outperform comprehensive CMTs for IPA diagnosis but was superior to gomori methenamine stains for *P. jirovecii* pneumonia. By setting the clinical view as the final call of infection and comparing mNGS and simplex PCR of *P. jirovecii* with the diagnoses of the clinician, both the sensitivity and specificity of mNGS and the *P. jirovecii* PCR were very close from the previous study (12). In this cohort, with a paired analysis, no significant difference was found between the SDSMRN criterion and *P. jirovecii* PCR, but the *P. jirovecii* PCR showed its advantage over the RPM ratio criterion. Probably in the population with highly suspected *P. jirovecii* pneumonia, there was no need to use mNGS to look for a needle in a haystack (28). For viral detection, mNGS definitely exceeds the hypothesis of causative agents made by the clinician, which multiplex PCR is restricted to. However, novel emerging virus identification requires the annotated microbe genome reference, long read, and depth sequencing methods (29).

### Strengths

The majority of studies published by researchers evaluated the diagnostic performance of mNGS by setting up the final call for infection of clinicians as the golden standard. However, the mNGS performance in pathogen detection might have been disvalued if clinical impressions involved bias. By reviewing existing reports of both mNGS and CMTs from suspected severe pneumonia patients, this study utilized direct paired comparisons between mNGS and CMT to reveal whether these two methods were significantly different in causative pathogen detection. The study implied that mNGS performed better than fungi culture. Thus, clinicians who are faced with immunocompromised populations or who are working in less-developed areas without access to simplex fungus PCRs or antigen tests are suggested to order mNGS for LRT specimens.

It was also discovered that RPM could be widely used for mNGS results interpretation among clinicians and that the RPMsample/RPM_NTC_ ratio criterion outperformed regardless of pathogen categorizing.

### Limitations

The research was clearly limited in several ways. Not all samples from this cohort had a PCR test of the suspected viral pathogen. The mNGS performance in detecting common viruses responsible for LRT infection was only evaluated in a small proportion of patients due to the need to compare with available PCR results. This could impact less in a future prospective study with all samples sent for both mNGS and PCR screens. Also, the criteria that were selected in identifying positive microbes reported by mNGS were documented ones from previous studies. It would be better to come up with a threshold based on the quantity of sequenced species under the circumstance that quantitative PCR was run to validate those in each sample. Lastly, effects on the clinical decision of mNGS results were not discussed, which would be better evaluated in a randomized controlled trial.

## Conclusions

The RPM ratio criterion performed better with a higher accuracy for bacteria, fungi, and viruses than the SDSMRN criterion. By choosing positive criteria wisely, mNGS may contribute to revealing LRT infection etiology in hospitalized groups of potential fungal infections and in situations with less access to the multiplex PCR of LRT samples from the laboratory.

## Data Availability Statement

The datasets presented in this study can be found in online repositories. The names of the repository/repositories and accession number(s) can be found in the article/Supplementary Material.

## Ethics Statement

The studies involving human participants were reviewed and approved by Ethic Committee of Peking Union Medical College Hospital. Written informed consent for participation was not required for this study in accordance with the national legislation and the institutional requirements.

## Author Contributions

HQin drafted the paper. JP, LL, JW, LP, XH, MH, HQiu, and BD contributed to the data collection. All authors contributed to the article and approved the submitted version.

## Funding

This work was supported by Ministry of Science and Technology of China [Grant number: 2020YFC0841300].

## Conflict of Interest

The authors declare that the research was conducted in the absence of any commercial or financial relationships that could be construed as a potential conflict of interest.

## Publisher's Note

All claims expressed in this article are solely those of the authors and do not necessarily represent those of their affiliated organizations, or those of the publisher, the editors and the reviewers. Any product that may be evaluated in this article, or claim that may be made by its manufacturer, is not guaranteed or endorsed by the publisher.
