# Supplementary material for: A Retrospective Paired Comparison Between Untargeted Next Generation Sequencing and Conventional Microbiology Tests With Wisely Chosen Metagenomic Sequencing Positive Criteria
Source: Front Med (Lausanne). 2021 Oct 6;8:686247. doi: 10.3389/fmed.2021.686247 (PMC8526841; doi:10.3389/fmed.2021.686247)
Supplement: Supplementary file 1 [file Data_Sheet_1.docx]

Supplementary table 1 routinely performed conventional microbiology tests in our participating centers

| Pathogen | Technique | Sample | | Microbiological Tests | |
| --- | --- | --- | --- | --- | --- |
| Bacteria | Bacterial culture | | LRT sample | | Bacterial culture on appropriate media with bacterial identification by MALDI-TOF mass spectrometry |
|  | Smear | | LRT sample | | Acid-fast staining for *Mycobacterium tuberculosis* |
|  | Multiplex PCR | | LRT sample | | *Legionella pneumophilia* |
| Viruses | Multiplex PCR | | LRT sample | | Influenza A/B, human rhinovirus, adenovirus, respiratory syncytial virus (RSV), human metapneumovirus (HMPV), cytomegalovirus (CMV) |
| Fungi | Smear | | LRT sample | | India ink staining for *Cryptococcus* |
|  |  | |  | | Gomori methenamine stain for *Pneumocystis jirovecii* |
|  | Fungal culture | | LRT sample | | Fungal culture on appropriate media with fungal identification by MALDI-TOF mass spectrometry |
|  | Antigen detection | | BAL | | Galactomannan |
|  | Multiplex PCR | | LRT sample | | *Pneumocystis jirovecii* |
| Others | Culture | | LRT sample | | *Nocardia* spp. |
|  | Multiplex PCR | | BAL | | *Mycoplasma pneumoniae*, *Chlamydia pneumoniae* |

LRT: lower respiratory tract; BAL: bronchoalveolar lavage fluid; MALDI-TOF, matrix-assisted laser desorption/ionization-time of flight

Supplementary table 2 positive agreement of conventional microbiology test results

| Pathogen | Definition of Clinically Significant Microbes |
| --- | --- |
| Bacteria | Moderate to heavy growths of bacteria with few epithelial cells seen on gram stain examination (< 10 per high power field) from LRT sample;  Oral commensal organisms considered as contaminants unless they are deemed significant by physician |
| *Legionella*, *Mycoplasma*, *Chlamydia* spp. | Positive PCR test for *Legionella*, *Mycoplasma*, *Chlamydia* spp. from LRT sample |
| *Mycobacterium tuberculosis* or NTM | Positive culture, anti-fast staining, or PCR test for *Mycobacterium tuberculosis* or NTM from LRT sample |
| *Aspergillus* spp. | Positive culture from LRT sample, or BAL GM > 1.0, or serum GM > 0.5 |
| *Pneumocystis jirovecii* | Detection of cyst by Gomori methenamine stain, or positive PCR for *Pneumocystis jirovecii* from LRT sample |
| Other fungi | Positive culture for fungi other than *Aspergillus* spp. from LRT sample; *Candida* spp. disregarded unless they are deemed significant by physician |
| *Cryptococcus* | Detection of capsule by India ink stain, positive culture or positive antigen test for *Cryptococcus* from LRT sample; |
| Cytomegalovirus | ≥10^4^ copies/ml by quantitative PCR from LRT sample |
| Other viruses | Positive PCR test for virus of interest from LRT sample |

LRT: lower respiratory tract; BAL: bronchoalveolar lavage fluid; MALDI-TOF, matrix-assisted laser desorption/ionization-time of flight

Supplementary table 3 Performance of metagenomic sequencing for specific pathogen detection

|  | RPM_sample_/RPM_NTC_ ratio ≥ 10/1 | | | | |  | |  | SDSMRN ≥ 3/1 | | | |  | | |  |  |  |
| --- | --- | --- | --- | --- | --- | --- | --- | --- | --- | --- | --- | --- | --- | --- | --- | --- | --- | --- |
|  | Sensitivity | Specificity | PPV | | NPV | | Accuracy | | Sensitivity | Specificity | PPV | | NPV | Accuracy | | | |  |
| *Acinetobacter baummannii* | | 56%  (35.3%-75.0%) | 87.1%  (79.6%-92.2%) | | 46.7%  (28.8%-65.4%) | 90.8%  (83.7%-95.1%) | | 81.9%  (75.7%-88.1%) | | 76%  (54.5%-89.8%) | 74.2%  (65.4%-81.4%) | | 37.3%  (24.5%-51.9%) | 93.9%  (86.6%-97.5%) | | 74.5%  (67.5%-81.5%) | | |
| *Pseudomonas*  *aeruginosa* | | 77.8%  (40.2%-96.1%) | 95%  (89.6%-97.8%) | | 50%  (24.0%-76.0%) | 98.5%  (94.2%-99.7%) | | 94.0%  (90.1%-97.8%) | | 77.8%  (40.2%-96.1%) | 87.9%  (81.0%-92.6%) | | 29.2%  (13.4%-51.2%) | 98.4%  (93.8%-99.7%) | | 87.2%  (81.9%-92.6%) | | |
| *Klebsiella*  *pneumonia* | | 58.3%  (28.6%-83.5%) | 99.3%  (95.4%-100.0%) | | 87.5%  (46.7%-99.3%) | 96.5%  (91.5%-98.7%) | | 96.0%  (92.8%-99.1%) | | 66.7%  (35.4%-88.7%) | 94.2%  (88.4%-97.3%) | | 50.0%  (25.5%-74.5%) | 97.0%  (92.0%-99.0%) | | 91.9%  (87.6%-96.3%) | | |
| *Staphylococcus*  *aureus* | | 40%  (7.3%-83.0%) | 98.6%  (94.6%-99.8%) | | 50%  (9.2%-90.8%) | 97.9%  (93.6%-99.5%) | | 96.6%  (93.8%-99.5%) | | 80%  (29.9%-98.9%) | 97.2%  (92.6%-99.1%) | | 50%  (17.4%-82.6%) | 99.3%  (95.5%-100%) | | 96.6%  (93.8%-99.5%) | | |
| *Stenotrophomonas maltophilia* | | 75%  (21.9%-98.7%) | 98.6%  (94.6%-99.8%) | | 60%  (17.0%-92.7%) | 99.3%  (95.6%-100%) | | 98.0%  (95.7%-100%) | | 75%  (21.9%-98.7%) | 97.2%  (92.6%-99.1%) | | 42.9%  (11.8%-79.8%) | 99.3%  (95.5%-100%) | | 96.6%  (93.8%-99.5%) | | |
| Cytomegalovirus | | 100%  (51.7%-100%) | 83.3%  (68.0%-92.5%) | | 46.2%  (20.4%-73.9%) | 100%  (87.7%-100%) | | 85.4%  (75.4%-95.4%) | | 100%  (51.7%-100%) | 69.0%  (52.8%-81.9%) | | 31.6%  (13.6%-56.5%) | 100%  (85.4%-100%) | | 72.9%  (60.3%-85.5%) | | |
| Influenza | | 66.7%  (12.5%-98.2%) | 100%  (77.1%-100%) | | 100%  (19.8%-100%) | 94.4%  (70.6%-99.7%) | | 95%  (85.4%-100%) | | 66.7%  (12.5%-98.2%) | 100%  (77.1%-100%) | | 100%  (19.8%-100%) | 94.4%  (70.6%-99.7%) | | 95%  (85.4%-100%) | | |

NPV, negative predictive value; PPV, positive predictive value


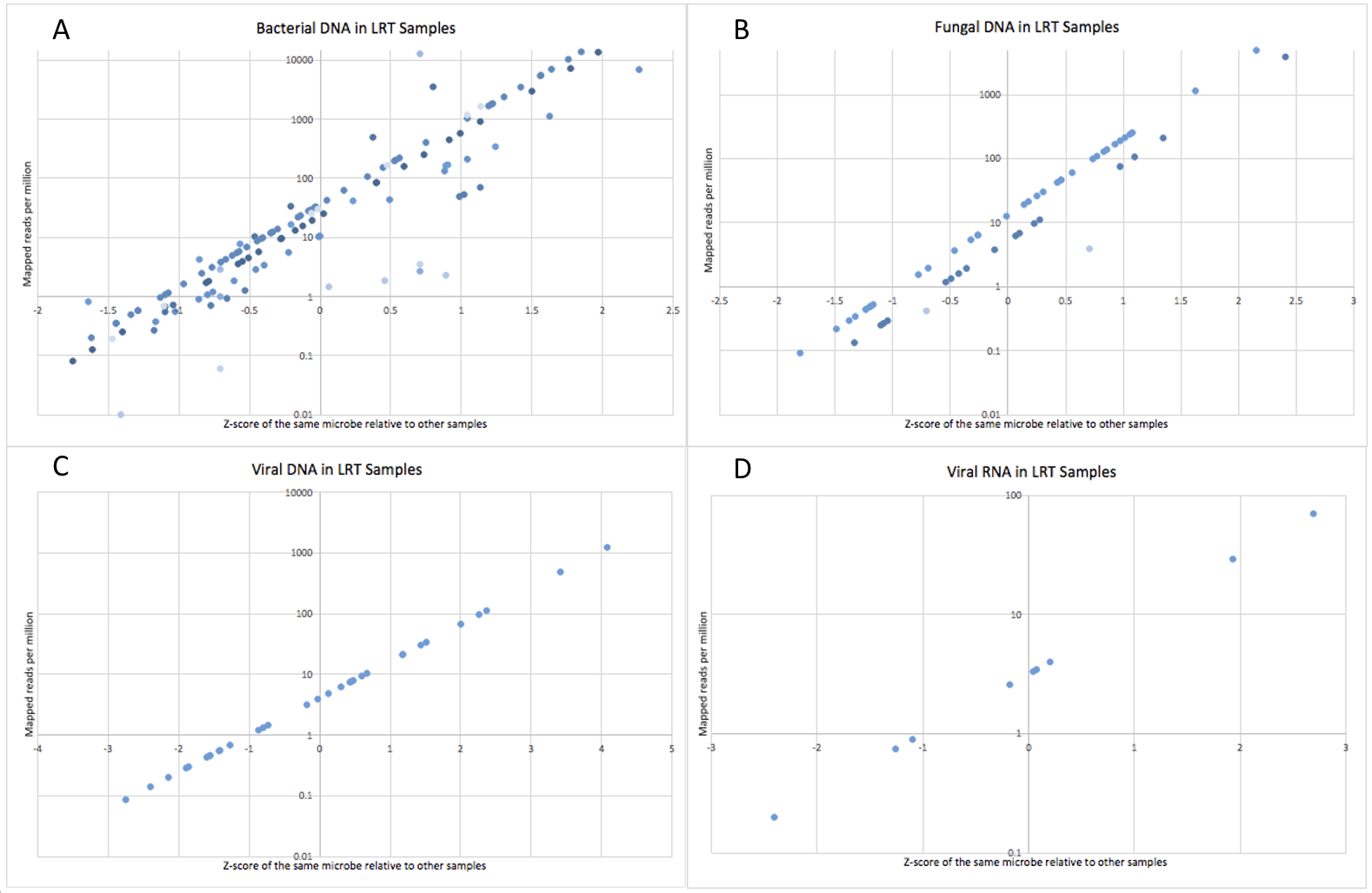


Supplementary figure 1 Mapped reads abundance relative to each species in other samples of our cohort using Z-score
